# Inquiry into the Condition of the Medical College of Ohio

**Published:** 1833

**Authors:** 


### VII. Inquiry into the Condition of the Medical College of Ohio.

In our last number, we inserted several of public documents, relative to alleged abuses in the government and organization of the Medical College of Ohio; one of which, a resolution of the General Assembly, provided for the appointment of a Court of Inquiry by the Governor. The gentlemen appointed, met "according to law" on the 2nd Monday of April, and continued in session nearly three weeks. J. W. Willey, Esq. of Cleaveland, was appointed Chairman, and Dr. Z. M. Krider, of New Lancaster, Secretary. A long catalogue of charges and specifications were received by the Court, from Dr. Joshua Martin, of Xenia, President of the late Third District Medical Society of Ohio, and more than 50 witnesses were examined under oath. E. P. Cranch, Esq. of the Cincinnati Bar, acted as stenographer, and so voluminous was the testimony, that, we are informed, he has not yet completed a copy of it for the use of the Court; which is to meet in the course of the present summer, at Columbus, to examine it and prepare a report to the Legislature. It is to hoped, this report will be accompanied with such recommendations, as may guide that honorable body into a course of legislation, calculated to suppress the opposition which now prevails against the school, and raise it to a higher rank among our medical institutions. Should they do this they will deserve the thanks of the public.

